# Extraskeletal Myxoid Chondrosarcoma: Long-Term Survival in the Setting of Metastatic Disease

**DOI:** 10.1155/2020/2684746

**Published:** 2020-09-05

**Authors:** Karim Masrouha, Iqbal Multani, Om Bhatt, Michelle Ghert

**Affiliations:** ^1^Hamilton Health Sciences, Juravinski Hospital and Cancer Centre, 711 Concession St, Hamilton, ON, Canada L8V 1C3; ^2^Centre for Evidence-Based Orthopaedics, Department of Surgery, McMaster University, 1280 Main St W, Hamilton, ON, Canada L8S 4L8; ^3^The Royal North Shore Hospital, Northern Sydney Local Health District, Douglas Building, Level 5 Reserve Rd, St Leonards NSW 2065, Australia; ^4^Faculty of Engineering and Faculty of Health Sciences, McMaster University, 1280 Main St W, Hamilton, ON, Canada L8S 4L8

## Abstract

Three cases of extraskeletal myxoid chondrosarcoma (EMC) in patients who presented with pulmonary metastases and were managed with long-term close observation without systemic intervention are presented. Follow-up imaging showed slow progression of their disease over several years, and the patients remained asymptomatic from their pulmonary metastases. This clinical experience provides insight into the natural history of the disease and suggests that some patients may experience long-term survival and remain asymptomatic even without systemic intervention, thereby improving their quality of life by avoiding potentially debilitating treatments.

## 1. Introduction

Extraskeletal myxoid chondrosarcoma is a rare malignant sarcoma affecting mesenchymal tissue; it comprises less than 3% of soft tissue tumors [1]. It is believed to be caused by chromosomal translocations resulting in a gene fusion, typically t(9;22)(q22;q12.2), fusing EWSR1 to NR4A3 [2]. This translocation can disrupt the transcriptional regulation of specific target genes, resulting in neoplasia [3]. These tumors typically have an indolent course, although they have been known to recur and/or metastasize over 10 years following the initial diagnosis, suggesting an intermediate-grade neoplasm [4]. Despite this, the overall survival of EMC has been reported to be as high as 100% after 5 years and 88% after 10 years [4]. The mainstay of treatment is wide surgical resection, as radiation therapy and chemotherapy are not considered to be effective [5]. Notwithstanding, most patients who present with systemic disease (pulmonary metastases) are treated with a combination of these modalities [4, 6, 7]. Given the inherent risks of these treatments and the lack of any clear benefit, close observation is a little-discussed management method that could be an option offered to patients with EMC who present with metastatic disease.

## 2. Case Presentation

### 2.1. Case 1

A healthy 48-year-old male patient, nonsmoker, presented five months after noticing a lump in his gluteal cleft. He had no pain. The lump was noticed after significant intentional weight loss. The mass was subcutaneous, soft, nontender, and mobile on examination. It measured approximately 3 × 3 cm. There were no overlying skin changes. His lower extremities were neurovascularly intact.

**Figure 1:**
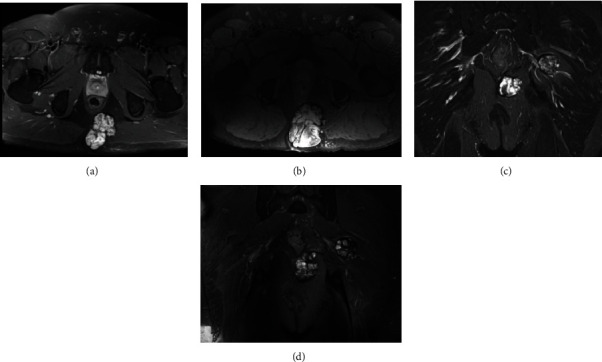
Initial (a, c) and 3-year follow-up (b, d) axial and coronal T2-weighted magnetic resonance imaging of the pelvis (Patient 1), demonstrating both the primary mass and the satellite lesion.

Magnetic resonance (MR) imaging showed a lobulated mass around, but not invading, the coccyx and a second mass at the level of the left sciatic notch, deep to the gluteus maximus muscle (Figure 1). A bone scan was negative for bone metastasis. Computed tomography (CT) of the chest showed innumerable pulmonary nodules. An ultrasound-guided core needle biopsy of the palpable gluteal cleft mass confirmed EMC by histology and molecular studies, including abnormal fluorescence in situ hybridization (FISH) for the EWSR1 gene rearrangement confirming the diagnosis [8].

The patient underwent clinical examinations and chest imaging every three months for the first year and every six months thereafter. He did not undergo any local management of his primary tumor. No systemic treatment was received. The patient had significant medical comorbidities, which precluded consideration for treatment with chemotherapy.

The patient was followed for 50 months. He demonstrated slow progression of his pulmonary nodules over time. The patient has not had any pulmonary symptoms nor has he had any alteration in his activities of daily living. The patient experienced slow progression of his primary mass and has developed some occasional discomfort with prolonged sitting due to its location (coccyx).

### 2.2. Case 2

A 63-year-old male patient, nonsmoker, was found to have lung nodules on chest imaging which was performed prior to insertion of an Implantable Cardioverter Defibrillator (ICD) for hypertrophic cardiomyopathy. Investigation revealed the presence of a left lateral thigh mass. He had not noticed it previously, and it was not painful. The mass was overlying the tensor fascia lata in the proximal thigh and was firm, nontender, and mobile on examination. It measured 5 × 6 cm. His lower extremities were neurovascularly intact.

CT images of the left thigh showed a soft tissue mass within the tensor fascia lata muscle. CT of the chest confirmed the presence of multiple pulmonary nodules. An ultrasound-guided core needle biopsy of the mass confirmed EMC by histology and molecular studies, including abnormal FISH for the EWSR1 gene rearrangement.

The patient underwent clinical examinations and chest imaging every three months for the first year and every six months thereafter. He did not undergo any local management of his primary tumor. No systemic treatment was received.

The patient was followed for 70 months. He demonstrated slow progression of his pulmonary nodules over time. The patient has not had any pulmonary symptoms nor has he had any alteration in his activities of daily living. The patient has not noticed any significant change in the size of his primary mass.

### 2.3. Case 3

A 65-year-old male, nonsmoker, presented with a four-month history of a right ankle lump that had grown in size and was associated with pain and discomfort on physical activity. He was known to have restrictive cardiomyopathy, obstructive sleep apnea, morbid obesity, asthma, hypertension, type II diabetes mellitus, and hypercholesterolemia. The mass was over the right lateral malleolus and was soft, nontender, and fixed on examination. It measured approximately 4 × 4 cm. His right foot was neurovascularly intact.

MR imaging showed a lobulated soft tissue mass around the right ankle, with both intra- and extra-articular components. Chest radiographs and CT images revealed innumerable pulmonary nodules. An open biopsy confirmed EMC by histology and molecular studies, including abnormal FISH for the EWSR1 gene rearrangement.

**Figure 2:**
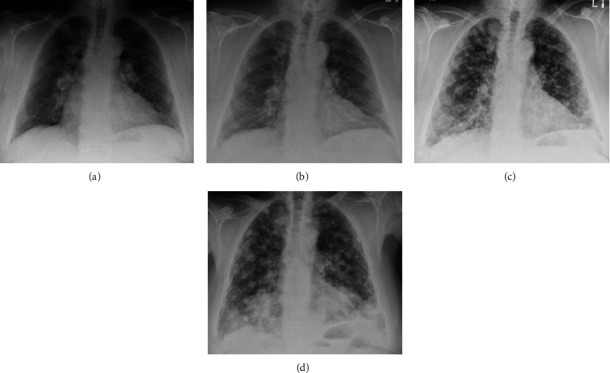
Chest radiographs of Patient 3 showing slow progression of metastatic nodules at two years (a), five years (b), seven years (c), and 10 years (d) of follow-up.

The patient underwent clinical examinations and chest imaging every three months for the first year and every six months thereafter (Figure 2). He initially underwent a palliative below-knee amputation for his primary tumor as it was large, invading the ankle and subtalar joint, and was fungating through the skin. No systemic treatment was received. The patient had significant medical comorbidities, which precluded consideration for treatment with chemotherapy.

The patient was followed for 126 months, after which he died from complications of unresectable colon cancer. The patient reported shortness of breath with activity at his 10-year follow-up visit. This remained unchanged at this last visit and was being managed by a respiratory therapist. He sustained a pathologic right hip fracture prior to his death, which was treated with a hemiarthroplasty, though he remained wheelchair bound after that. Tissue sent for examination during right hip fracture fixation was consistent with EMC (Figure 2).

## 3. Discussion

This small case series elucidates the potential of managing EMC by nonsystemic methods, solely utilizing close clinical and radiographic observations. Supporting the findings in this report is one published case report of a patient with EMC who developed multiple pulmonary metastases after initial resection of their primary tumor and a solitary pulmonary nodule. This patient was followed with close observation for four years, remaining asymptomatic with slow progression of their pulmonary nodules [9]. Despite the lack of effectiveness of systemic therapies in treating EMC, patients who present with metastatic disease are often managed with a range of chemotherapeutic agents and radiation therapy, which can carry severe side effects and possess an inherent morbidity [4, 7]. Although the current report is a small cohort with no comparison group, it demonstrates the potential of nonsystemically managing certain patients who present with metastatic EMC, integrating long-term event-free survival and a high quality of life.

## 4. Learning Points/Take-Home Messages

1. EMC does not respond well to currently available systemic treatments
2. Patients with metastatic EMC may experience slow progression of their disease and remain asymptomatic in the long term, even without systemic intervention
3. These patients may be considered candidates for close observation, avoiding the potential morbidity of systemic treatments and thus improving quality of life

## 5. Patient's Perspective

I am a person who has lived with cancer for almost six years. I discovered this through a medical examination. At that time, I was given one year to live and advised to live the remainder of my life “to the fullest.” The diagnosis was a shock to both me and my wife. I had felt no ill effects from the cancer, but seeing the lung involvement on a television screen was a real eye-opener.

After the initial diagnosis, I was referred to the cancer center for treatment follow-up with the resident oncologist. After many CT scans and follow-up interviews, I began to feel that I could somehow control this disease.

My immune system began to kick in and I was told that although the cancer did not stop growing, the immune system was keeping the growth to a minimum. I am still going to the cancer center for an X-ray and follow-up every six months. I would like to thank the doctors at the cancer clinic for their help and guidance. I hope to enjoy many more years of life because of their professional assistance.
